# Frequent Toggling between Alternative Amino Acids Is Driven by Selection in HIV-1

**DOI:** 10.1371/journal.ppat.1000242

**Published:** 2008-12-19

**Authors:** Wayne Delport, Konrad Scheffler, Cathal Seoighe

**Affiliations:** 1 Institute of Infectious Disease and Molecular Medicine, University of Cape Town, Rondebosch, Cape Town, South Africa; 2 Centre for High-Performance Computing, Rosebank, Cape Town, South Africa; 3 Computer Science Division, Department of Mathematical Sciences, University of Stellenbosch, Stellenbosch, South Africa; University of Pennsylvania School of Medicine, United States of America

## Abstract

Host immune responses against infectious pathogens exert strong selective pressures favouring the emergence of escape mutations that prevent immune recognition. Escape mutations within or flanking functionally conserved epitopes can occur at a significant cost to the pathogen in terms of its ability to replicate effectively. Such mutations come under selective pressure to revert to the wild type in hosts that do not mount an immune response against the epitope. Amino acid positions exhibiting this pattern of escape and reversion are of interest because they tend to coincide with immune responses that control pathogen replication effectively. We have used a probabilistic model of protein coding sequence evolution to detect sites in HIV-1 exhibiting a pattern of rapid escape and reversion. Our model is designed to detect sites that toggle between a wild type amino acid, which is susceptible to a specific immune response, and amino acids with lower replicative fitness that evade immune recognition. Through simulation, we show that this model has significantly greater power to detect selection involving immune escape and reversion than standard models of diversifying selection, which are sensitive to an overall increased rate of non-synonymous substitution. Applied to alignments of HIV-1 protein coding sequences, the model of immune escape and reversion detects a significantly greater number of adaptively evolving sites in *env* and *nef*. In all genes tested, the model provides a significantly better description of adaptively evolving sites than standard models of diversifying selection. Several of the sites detected are corroborated by association between Human Leukocyte Antigen (HLA) and viral sequence polymorphisms. Overall, there is evidence for a large number of sites in HIV-1 evolving under strong selective pressure, but exhibiting low sequence diversity. A phylogenetic model designed to detect rapid toggling between wild type and escape amino acids identifies a larger number of adaptively evolving sites in HIV-1, and can in some cases correctly identify the amino acid that is susceptible to the immune response.

## Author Summary

Viruses, such as HIV, are able to evade host immune responses through escape mutations, yet sometimes they do so at a cost. This cost is the reduction in the ability of the virus to replicate, and thus selective pressure exists for a virus to revert to its original state in the absence of the host immune response that caused the initial escape mutation. This pattern of escape and reversion typically occurs when viruses are transmitted between individuals with different immune responses. We develop a phylogenetic model of immune escape and reversion and provide evidence that it outperforms existing models for the detection of selective pressure associated with host immune responses. Finally, we demonstrate that amino acid toggling is a pervasive process in HIV-1 evolution, such that many of the positions in the virus that evolve rapidly, under the influence of positive Darwinian selection, nonetheless display quite low sequence diversity. This highlights the limitations of HIV-1 evolution, and sites such as these are potentially good targets for HIV-1 vaccines.

## Introduction

Intra-host HIV evolution is characterized by very rapid escape from immune responses [1]–[5]. Such host immune selection pressures are typically mediated by neutralizing antibodies [6], T-helper cells [7] or Cytotoxic T Lymphocytes (CTLs) [1],[8],[9]. Escape mutations associated with neutralizing antibodies [10],[11] tend not to have a significant effect on the fitness of the virus [10], or rate of disease progression [12]. Many examples of CTL escape mutants are known, however, that affect both viral replication ability, and thus viral load [13]–[16], and rate of disease progression [17]–[20]. CTLs recognize viral epitopes bound by human leukocyte antigens (HLAs) at the surface of infected cells, causing cell death. The cellular processes by which CTL epitopes are cleaved and presented at the cell surface provide numerous opportunities for escape from the immune response. Escape can occur through viral mutations that affect proteosome processing, affinity for transport antigen processing (TAP) proteins, translocation of peptides to the endoplasmic reticulum, antigen processing prior to presentation, binding of MHC class I molecules and finally recognition by cytotoxic T cells [1]. Much of the work on immune escape from CTL responses in HIV-1 has focused on identifying escape mutations which either prevent MHC binding or recognition by CTLs [1],[2],[9],[19],[21],[22].

The effect of within-host HIV-1 evolution and immune escape on viral genetic variation at the host population level is highly topical. Early research indicating a strong association between HLA type and viral polymorphisms across individuals [23] was criticized for not adequately addressing population founder effects [4],[24]. Nonetheless, more recent studies, which account for spurious association of HLA alleles with viral polymorphisms resulting from shared ancestry, confirm widespread association between HLA alleles and polymorphisms in the viral amino acid sequence, illustrating the extent to which the virus adapts to the host-specific CTL response [4], [24]–[26].

Escape mutations from specific HLA-mediated immune responses that incur a cost to the virus in terms of replication fitness are thought to come under selective pressure to revert to wild-type upon transmission to a host lacking the immune response [2], [21], [25], [27]–[31]. Whether and how rapidly reversion occurs depend on both the fitness cost of the escape mutation [30], [32]–[35], and the occurrence of compensatory mutations that offset this cost [15],[22]. Escape from a host-immune response that occurs at a substantial fitness cost to the virus and thus reverts rapidly, can result in a pattern of switching or toggling [30],[31] between the amino acid which is most fit in the absence of the immune response (we refer to this as the wild type state) and amino acids that prevent CTL recognition (the escape state).

Models of coding sequence evolution have frequently been applied to HIV-1 sequences with the aim of identifying adaptively evolving sites [9], [36]–[40]. These models are designed to detect an elevation in the rate of non-synonymous substitution (*dN*) over the rate of synonymous substitution (*dS*), the latter being assumed to occur at the neutral rate of evolution. This is referred to as diversifying selection and it occurs when, on average, non-synonymous mutations result in an increase in fitness and thus have a higher fixation probability and a shorter fixation time than neutral mutations. In the idealized scenario, assumed by models of diversifying selection, all non-synonymous substitutions benefit from this increased rate, resulting in rapid diversification from the ancestral amino acid. Amino acid toggling, driven by positive selection to escape from immune responses and to revert to wild type in their absence is not specifically envisaged by these models.

We propose a model of positive selection associated with immune escape and reversion, which we call the toggling selection model. Our motivation is twofold. Firstly, the toggling model is significantly more realistic than the diversifying selection model in the context of selection associated with immune escape and reversion and, consequently, it is likely to have more power to detect positive selection associated with this process than a model of diversifying selection. Since immune escape and reversion are likely to be a common source of adaptive evolution in viral sequences, we hypothesized that the toggling model may have greater power, overall, to detect adaptively evolving sites. Second, although many previous studies have identified adaptively evolving sites in HIV-1 [9], [36]–[40], most have not been concerned with the patterns of sequence changes found at these sites, and none has attempted to distinguish systematically between adaptively evolving sites consistent with immune escape and reversion and sites evolving under diversifying selection.

Using simulation as well as publicly available HIV-1 data we compared the power of the toggling model and standard models of adaptive evolution to detect adaptively evolving sites. Because the real sequences were obtained from individuals with known HLA in which associations between HLA alleles and sequence polymorphisms had been established [4], we could investigate the relationship between the sites detected and sites that are putatively involved in adaptation to the host HLA type. We also used model comparison techniques to compare the fit of the toggling selection model to the fit of standard selection models in order to determine which model provided a better explanation of the HIV-1 data.

## Methods

### The Model

Probabilistic models of codon sequence evolution [41],[42] use a continuous-time Markov process, described by a rate matrix, *Q*, with element *q_ij_* denoting the instantaneous substitution rate from codon *i* to codon *j*. Here we introduce a novel variant of the codon model, designed to describe host-mediated immune escape from and reversion to a wild type amino acid *W*. The instantaneous rate matrix describing this model is
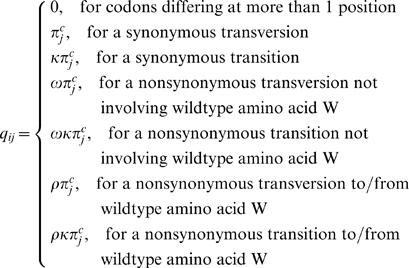
(1)where *κ* is the transition-transversion rate ratio, *ω* is the non-synonymous substitution rate relative to the rate of synonymous substitution (*dN/dS*) for substitutions not involving the wild type amino acid *W*, and *ρ* is the relative substitution rate for non-synonymous substitutions involving wild type amino acid *W.*

**Figure 1:**
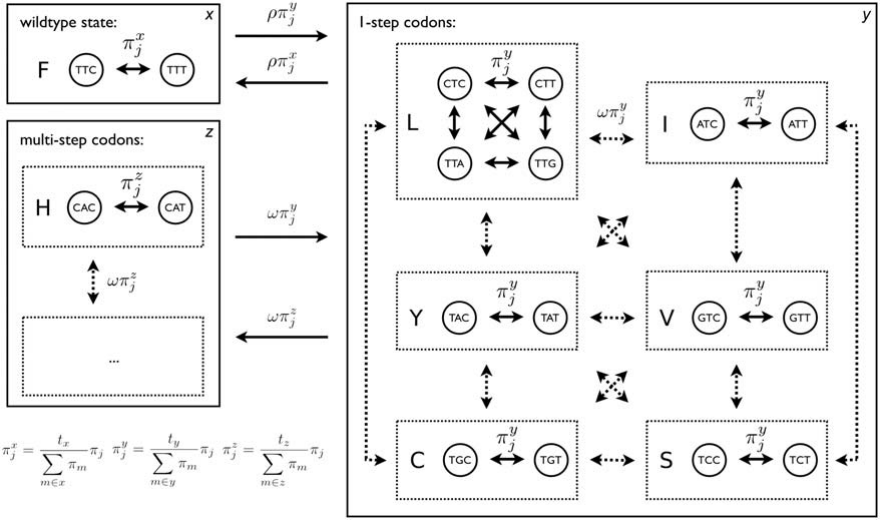
Codon model of amino acid toggling. The immune escape and reversion model has three classes of codons: codons encoding the wild type amino acid (class x); codons separated from the wild type by a single nucleotide substitution (y); and codons separated from the wild type by more than one substitution (z). In the example shown, phenylalanine (F) is the wild type amino acid. Rates of substitution between F and each of the six amino acids within one nucleotide substitution of F or from these amino acids back to F are affected by the parameter *ρ*, the amino acid toggling rate. All other non-synonymous substitutions have a multiplier *ω* instead of *ρ*. Rates of all substitutions depend on the frequency parameters 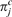, where *c* represents the codon class. The 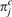 parameters take account of the codon bias estimated across the entire alignment and free parameters *t_c_* which describe the proportion of time spent by the site in each of the three codon classes.

For a given wild type state the 61 sense codons are divided into classes, *c*, depending on whether they encode the wild type amino acid (*c* = *x;* Figure 1), are separated from the wild type amino acid by a single nucleotide substitution (*c* = *y;* Figure 1), or are separated from the wild type by multiple substitutions (*c* = *z;* Figure 1). These codon classes are introduced to allow us to model the case in which immune escape and reversion involves repeated mutation from the wild type to an escape state, accessible to the wild type by a single nucleotide substitution, and back again. We introduce parameters, *t_c_*, to model the proportion of time the site spends in each class. The equilibrium frequency of a specific codon, belonging to class *c*, is then
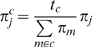
(2)where *π_j_* is the alignment-wide frequency of codon *j*, estimated using the *F3x4* model [41]. This formulation allows us to retain terms accounting for general codon usage bias, assumed to be shared across all sites in the model.

The model is fitted to the data one codon site at a time. Given a phylogenetic tree, the likelihood of the data at a site can be calculated using Felsenstein's pruning algorithm [43]. Because the identity of the wild type amino acid is unknown, we sum over all twenty amino acids such that
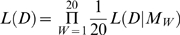
(3)where *L(D|M_W_)* is the likelihood of the data given that *W* is the wild type. As an alternative to summing over the uncertainty in the wild type state we can assume that the most common amino acid at a given site is the wild type. We tested both of these approaches and refer to them as the toggling and consensus toggling models, respectively.

Our test of positive selection comprises the comparison of log likelihoods between a null model, in which both the rate of amino acid toggling (*ρ*), and non-synonymous to synonymous substitution rate (*ω*) are constrained to be less than one, to an alternate model in which the constraint on the rate of amino acid toggling is removed. An alternative test involves the removal of both constraints (on *ρ* and *ω*), which we designate the unconstrained toggling model. The performance of the toggling models for detecting positive selection was evaluated and compared to existing approaches. In the latter, a site-specific diversifying selection model is defined in which the test of positive selection involves the comparison of log-likelihoods between a null model, for which *ω* is constrained to be less than one, to an alternate model in which the constraint is removed [44]. Null and alternate models are compared in all cases with a site-wise likelihood ratio test.

The above models require phylogenetic trees as input. In all cases the trees were estimated using *phyml* [45] under a general time-reversible model [46] with substitution rates modeled as a 4-category gamma distribution [47]. Branch lengths were fixed to maximum likelihood estimates obtained from the optimization of a nucleotide model using the entire alignment, but scaled with a nucleotide to codon scaling parameter, *R*, which was estimated separately for each site thus allowing site-wise variation in synonymous rates. To prevent spurious signals of selection resulting from recombination [48]–[50] we identified recombination breakpoints using GARD [51], and estimated phylogenies independently for each partition defined by these breakpoints.

### Simulation Strategy

We used simulations to evaluate the performance of the toggling model compared to the diversifying selection model to detect both diversifying selection and toggling selection. A phylogenetic tree inferred from a randomly selected subset of 100 taxa from a previously published *nef* gene alignment [4] was estimated as above. We simulated amino acid toggling and diversifying selection (200 codons each) for each of five parameter sets (Table 1), using custom scripts written in the HyPhy [52] batch language. Amino acid toggling (Figure 1) was simulated with variable values of *ρ* (Table 1), *ω = *0.05 for substitutions not involving the wild type state *W*, *t_x_* = 0.5, *t_y_* = 0.475, *t_z_* = 1−(*t_x_*+*t_y_*) = 0.025, such that most time was spent either in the wild type (*codon class x*) or codons separated from the wild type by a single nucleotide substitution (codon class *y*) (Figure 1). Diversifying selection was simulated across a range of different values of *ω* (Table 1).

**Table 1:** Power (%) to detect selection with alternative models.

A. Simulate Toggling				B. Simulate Diversifying Selection			
*ρ*	*T*	*T_(u)_*	*D*	*ω*	*T*	*T_(u)_*	*D*
2	14.5	6.5	8.5	1.1	1	3.5	7
3	29	19	9.5	1.7	8.5	20	25
4	37.5	25	11	2.3	10.5	27	38.5
5	41	31	21.5	2.8	17	36.5	61.5

*ρ,* non-synonymous to synonymous rate ratio associated with mutations away from or towards wild type amino acid; *ω,* non-synonymous to synonymous rate ratio for mutations not involving wild type; *T,* toggling model where *ρ* is unconstrained in alternate; *T_(u)_*, toggling model in which both *ρ* and *ω* are unconstrained; *D,* diversifying selection model.

We determined the effect of both tree length and tree shape on the detection of positive selection and toggling using simulations. We increased the size of the simulated data set to 200 randomly drawn taxa from a previously published *nef* alignment [4], effectively doubling the total tree length (Table 2). Trees inferred from HIV-1 sequences tend to have longer terminal branches. To investigate the effect of tree shape on power to detect selection we simulated data along a tree for which branch lengths were drawn randomly from an exponential distribution with mean = 0.05, using previously developed HyPhy code [44]. Power was calculated as the number of sites at which positive selection was correctly inferred as a proportion of all sites for which positive selection (either diversifying selection or toggling) was simulated. False positive rates, estimated as the number of sites at which positive selection was incorrectly inferred as a proportion of all sites not evolving under positive selection, was evaluated with simulations of 800 neutral and purifying selection codons (75% purifying, *ω* = 0.05; 25% neutral, *ω* = 1).

**Table 2:** The effect of tree length and shape on the power to detect amino acid toggling.

*ρ*	HIV-1 Tree (*n* = 100, *TL* = 13.8)		HIV-1 Tree (*n* = 200, *TL* = 26.5)		Random Tree (*n* = 100, *TL* = 14.2)	
	*T*	*D*	*T*	*D*	*T*	*D*
2	14.5	8.5	29.5	6	26.5	1
5	41	21.5	69	30	73	6.5

*ρ,* non-synonymous to synonymous rate ratio for mutations away from or towards wild type amino acid; *T,* toggling model where *ρ* is unconstrained in alternate; *D,* diversifying selection model.

### Application to Real Data

We obtained *gag*, *nef* and *pol* sequence alignments from previously published studies [4],[53], and an *env* alignment of HIV-1 subtype C sequences from the Los Alamos HIV databases (http://www.hiv.lanl.gov/content/index). Because we wished to make comparisons between results obtained on different genes it was important that the number of sequences in each alignment be approximately the same. However, we note that power is dependent on both the number of sequences and the amount of variation in those sequences. Since it is not possible to control both variables simultaneously in the real data we report the tree lengths in order to facilitate the comparison of results from different genes. We randomly sampled 100 sequences from each of the large *env, nef,* and *pol* genes and took all of the 98 sequences in the *gag* alignment [53]. The toggling selection model is more computationally intensive than the diversifying model, requiring 20 optimizations per codon site (one optimization for each wild type amino acid), for both the null and the alternative models. Use of a subset of the sequences in the larger alignments also helped to reduce running times. Sequences with stop codons within genes were pruned from the alignments. We used both a site-wise diversifying selection model and toggling selection model to identify adaptively evolving sites. For each gene we compared the fit of a toggling model versus diversifying selection model at each site using AIC [54]. Alignments used are available from the authors on request.

### Implementation

Both the model and simulation scripts have been implemented in the HyPhy [52] batch language and are available from the authors on request.

## Results

### Simulation Results

We have developed a model (Figure 1), designed to detect positive Darwinian selection associated with host-mediated immune response and reversion acting on viral protein-coding genes. Existing models of positive selection acting on coding sequences are sensitive to an overall elevation in the rate of non-synonymous to synonymous substitutions (we refer to this situation as diversifying selection here). Such a situation occurs when, on average, the effect on viral fitness of any amino acid-changing mutation is positive. For a class of viral sites that mediate escape from host immune responses at a cost to the virus in terms of replicative fitness, we do not necessarily expect an overall elevation in the rate of non-synonymous substitutions. Instead we expect to see switching between a wild type amino acid associated with high replicative fitness and susceptibility to the immune response, and an escape state with lower fitness. This model is motivated by the fact that, firstly the identification of sites that switch between wild type and escape states is of interest, because at these sites escape mutations are likely to be common, despite having a deleterious impact on viral fitness. Secondly, some sites with a rate of substitution between specific amino acids, which is higher than expected under neutrality may not have an overall rate of non-synonymous substitution greater than the neutral rate when we average over all possible non-synonymous substitutions.

**Figure 2:**
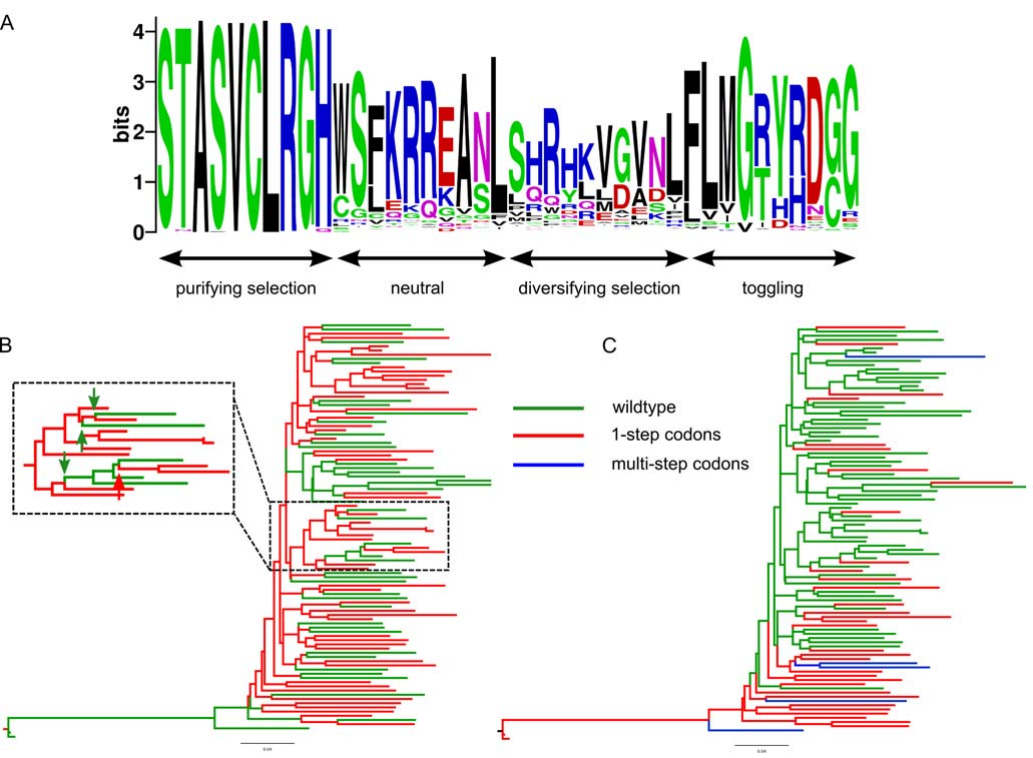
Simulation along HIV-1 *nef* phylogeny. Data was simulated under purifying selection, neutrality, diversifying selection, or toggling to evaluate power and false positives rates. (A) Amino acid sequence logos [73] of ten randomly drawn codon sites for each category of simulated site. (B) Simulated toggling site mapped to HIV-1 phylogeny showing the occurrence of escape (red arrows) and reversion (green arrows) mutations. (C) Simulated diversifying selection site mapped to HIV-1 phylogeny.

**Figure 3:**
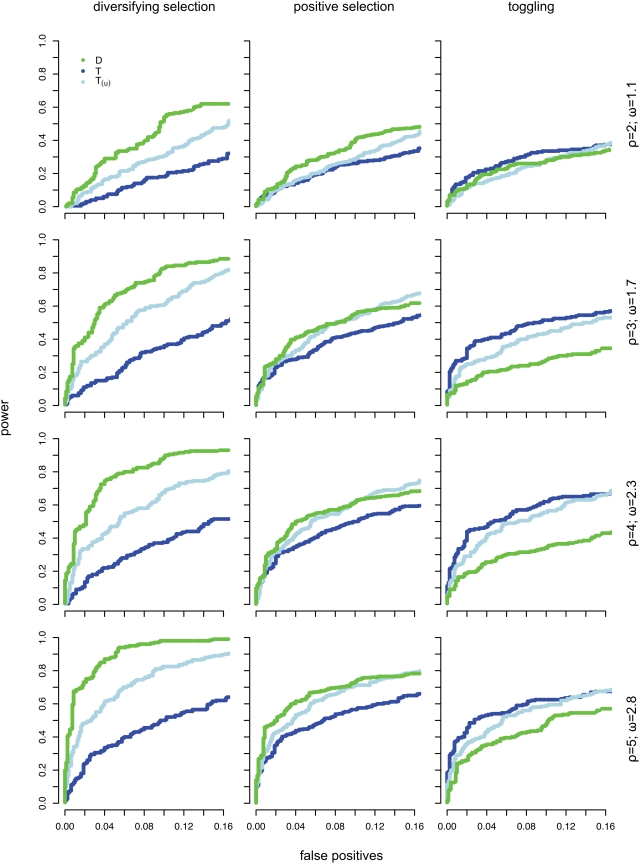
Evaluation of power and false positives. ROC curves indicating power and false positive rates for the detection of diversifying selection (left panel), diversifying selection and toggling (centre panel), and toggling (right panel) for each of the five parameter sets (Table 1) simulated.

Our test of positive selection involves the use of model comparison techniques to compare a null model in which the parameters *ρ* (describing the rate of immune escape and reversion relative to the synonymous substitution rate) and ω (the relative rate of all other non-synonymous substitutions) are constrained to be less than one, to an alternate model where *ρ* is unconstrained. Simulations (Figure 2) indicated improved power of the toggling model (*T*) over a standard diversifying selection model (*D*) (Table 1A, Figure 3, Table S1) to detect positive selection involving switching between a wild type state and escape states, consistent with host-mediated immune response. A diversifying selection model showed improved power to detect positive selection when data were simulated under a diversifying selection model (Table 1B, Figure 3, Table S1). We accounted for uncertainty in identifying the wild type state by averaging the likelihoods over all possible wild type amino acids at each codon (see Equation 3 in Methods). This would result in the loss of some power over a model in which the wild type state is known *a priori*. Given this loss in power associated with averaging over all potential wild type states in the toggling model, a model in which the wild type state at a site is assumed to be the same as the consensus amino acid at that site might be expected to have greater power. However, simulations indicated this model to have equivalent or less power (results not shown), suggesting that the consensus is not always a good approximation of the wild type state (the amino acid with highest replicative fitness in the absence of a specific immune response). Furthermore, a model in which the wild type state is taken from the data runs the risk of bias in model comparisons, because the model is defined using the data (wild type state taken to be the consensus in the actual data) and evaluated on the same data. As an alternative to averaging over all amino acids or drawing the wild type state from the consensus, we weighted the likelihoods for each potential wild type state by the observed alignment-wide amino acid frequency. However, this approach resulted in no increase in power and therefore we used equal weights on amino acids for the remainder of the analyses.

We also compared power when both the non-synonymous to synonymous rate ratio associated with the wild type state (*ρ*) and that of other non-synonymous substitutions (*ω*) are unconstrained (*T_u_*). Simulations confirmed that the model with both parameters unconstrained has greater power to detect diversifying selection (Table 1B) than the toggling model in which only *ρ* is unconstrained, but less power than a standard diversifying selection model (Table 1B). This loss of power against a diversifying selection model is due to the extra degree of freedom in the *T_u_* model, compared to both the diversifying selection and toggling models. For the same reason, the test in which both *ρ* and *ω* are unconstrained, has lower power to detect amino acid toggling (Table 1A) than the model in which only *ρ* is unconstrained. False positive rates at the 5% significance level were low for all models evaluated on a dataset consisting of a mixture of neutral and purifying selection sites (*D* = 0.88%; *T* = 1.63%; *T_u_* = 1.25%), and approximately equal to the expected rate of false positives when only neutral sites (*ω* = 1) were simulated (*D* = 3.3%; *T* = 5.6%; *T_u_* = 4.7%).

We found that the power to detect toggling increased dramatically with larger data sets and with trees with exponentially distributed branch lengths (Table 2, Figure S1A, Figure S2). Typical phylogenetic trees inferred from HIV-1 sequences have long terminal branches and pose a challenging problem for the toggling selection model. This is because escape and reversion events that occur on the same branch are not observed. The power of a diversifying selection model to detect positive selection involving toggling was much lower when data were simulated along a random tree and did not show much improvement with a larger dataset (Table 2). We also evaluated the power of the toggling selection model to recover the amino acid used as the wild type in simulation, and the proportion of time spent in each of the three codon classes. The amino acid which maximized the likelihood of the toggling selection model, was inferred to be the wild type. In simulations the wild type state was always identified correctly (100% success rate) for both intermediate (*ρ* = 2) and rapid (*ρ* = 5) rates of toggling, and the inferred time spent in each state was also estimated accurately (*t_x_*: simulated 0.5; inferred 0.494±0.128, *t_y_*: simulated 0.475; inferred 0.471±0.128).

### Real Data

**Figure 4:**
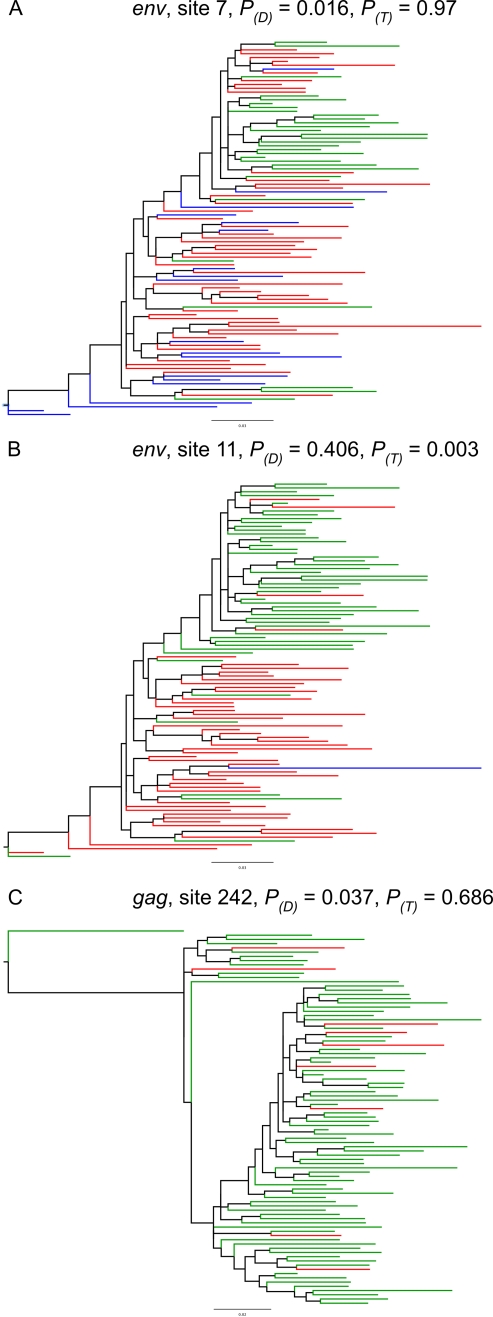
Evidence of toggling and diversifying selection in real data. Tree branches are coloured according to the codon category of the node at the right end of the branch for sites detected to be (A) diversifying selection or (B) toggling, and (C) a previously identified HLA-associated polymorphism in *gag* (TW10). Potential escape and reversion mutations are mapped as red and green arrows, respectively. *P,* likelihood ratio test statistic *p*-value; *D,* diversifying selection model; *T,* toggling model. Both *env* and *gag* trees are rooted on HIV-1 subtype B.

We used the toggling model developed above to detect putative escape-and-reversion sites in four HIV-1 datasets (see Methods; tree lengths: *nef* = 10.8, *gag* = 9.1, *env* = 17.3, *pol* = 8.6). Amino acid toggling is evident at sites at which multiple mutations away from the wild type and reversions back to wild type are observed (Figure 4). For all genes evaluated, the toggling selection model provided a better fit than a diversifying selection model for the majority of positively selected sites (Table 3). By contrast, when all sites are considered, the toggling model provides a better fit for a smaller proportion of sites (Table 3). The toggling model detected significantly more positively selected sites in *nef* and *env* (Binomial test, *nef*: *P* = 0.001374; *env*: *P* = 0.001028), than a standard diversifying selection model (Table 3, Figure S3). Neither diversifying selection sites nor toggling selection sites occurred more frequently than expected by chance within optimal HLA epitopes (Figure S4). Similarly there was no clear association between CTL-reactive peptides identified in *gag* [16] and amino acid toggling (*P* = 0.055; Figure S4).

**Table 3:** Fit of the diversifying selection compared to the toggling model for HIV-1 sequences.

	*AIC*_(*T*)_>*AIC*_(*D*)_		Number of Positively Selected Sites Detected			Total Sites
	D or T	All	*D or T*	*D Only*	*T Only*	
*pol*	0.69	0.13	13	9	8	486
*nef*	0.84	0.46	25	11	21	177
*gag*	0.65	0.23	29	18	19	496
*env*	0.92	0.55	58	32	42	439

*AIC,* Akaike Information Criterion; *T,* toggling model where *ρ* is unconstrained in alternate; *D,* diversifying selection model.

Because the mapping of optimal CTL epitopes to positively selected sites ignores population specific HLA frequencies and selective pressures, we evaluated our results for *nef* against HLA-associated polymorphisms detected in the same patient cohort [4]. The HLA associations detected on the full dataset (n = 684), and in this study, are shown in Table 4. Fifteen of the 84 codon sites for which a significant association between HLA allele and polymorphism was previously detected [4], were detected with the toggling selection method (using just 100 of the 684 sequences from which the associations were inferred). We found significant enrichment for HLA-associated polymorphisms among sites detected with the toggling model (*P* = 0.001544), or with a diversifying selection model (*P* = 0.008397). Since our model evaluates each of twenty amino acids as the candidate wild type state we were able to infer both the identity of the wild type amino acid at each site showing evidence of positive selection, and the proportion of time spent in the wild type versus escaped states (Table 4). More than half of the sites detected as toggling spend the majority of time at codons that either code for the wild type amino acid, or are a single nucleotide substitution away from the wild type. Sites with multiple wild type amino acids (e.g. sites 10 and 15) may be associated with multiple overlapping immune responses or with multiple equally fit amino acids. These sites were frequently also detected using a diversifying selection model. Many of the inferred wild type amino acids (at sites 33, 50, 54, 82, 83, 100, 101, 126, 178) were consistent with previously identified escape and reversion mutations identified from association with HLA alleles [4],[24].

**Table 4:** HLA allele associated polymorphisms [4] at detected toggling sites.

Codon	Site Composition	*WT*	E	R	*t_x_*	*t_y_*	HLA Alleles
3#	A_1_ G_86_ N_4_ S_4_	S			0.021	0.978	
6	S_96_	**S**			0.821	0.004	
10* #	A_4_ C_1_ E_1_ F_2_ G_8_ I_8_ K_1_ L_22_ M_12_ R_4_ S_4_ T_2_ V_20_ W_1_	**M**,L,A,V,S,P,T,Y,H,Q,K,E,C,W,R,G			0.086	0.413	
14*	A_7_ D_1_ H_2_ L_1_ N_1_ P_66_ Q_1_ S_13_ T_5_	**S**,P,H	Y		0.076	0.212	B08
15*	A_56_ D_1_ G_1_ H_1_ I_2_ K_7_ N_1_ Q_2_ R_4_ S_3_ T_14_ V_1_	**A**,S,T,H,N,K,D,E,R,G	D,T	A	0.034	0.005	A31, B51, B57
24	A_3_ D_2_ E_81_ G_2_ M_1_ P_1_ Q_3_ R_3_ T_1_	**T**,L,M,V,K,G	-	E	0.007	0.465	B54, C02, C06
26#	A_94_ Q_2_ R_3_	**P**,R			0.002	0.841	
33	A_51_ S_3_ V_46_	**A**,V	A	A,V	0.609	0.273	A11, A68
40*	A_1_ G_5_ H_74_ K_1_ R_6_ Y_8_	**H**,Q	R		0.021	0.024	B37
49	A_75_ N_9_ P_6_ S_1_ T_3_	**T**,D,H,K	R		0.001	0.998	B57
50	A_80_ E_2_ H_1_ I_3_ N_6_ S_2_ T_4_	**D**,L,P,T,Y	G,D,E	T,A	0.001	0.085	B14, B35, B53, B57, B58
54	A_30_ D_65_ E_1_ N_1_ T_2_	**A**,D	A	D	0.316	0.036	B14, C08
82	A_1_ G_2_ K_80_ Q_1_ R_4_ Y_2_	**A**,L,P,T	R	R,K	0.021	0.145	A03, B14, B15
83*	A_29_ E_1_ G_49_ K_2_	**G**,A	A,G	A,G	0.927	0.073	A03, A11, B15, B40, B44, B55, C03, C07
100	I_4_ L_86_ M_6_ Y_1_	**M**,I	M	I,L	0.213	0.075	A03, B40, C03
101*	H_1_ I_74_ P_2_ S_1_ T_1_ V_16_ Y_2_	**L**,I,T,A	I,V	V	0.004	0.830	B14, B40, C01, C08
103#	H_1_ K_1_ Q_3_ S_91_	**S**,I,T,R			0.995	0.002	
126	C_1_ G_2_ N_90_	**S**,Y,R,G	S,C	N	0.001	0.999	A26, B51, C14
170#	A_1_ C_4_ H_1_ L_1_ N_10_ Q_1_ S_74_	L			0.470	0.410	
178*	E_2_ G_14_ K_53_ R_25_	**R**,K	R	K	0.240	0.760	B40
182	E_12_ I_2_ K_5_ L_2_ M_19_ Q_22_ V_34_ W_3_	**L**,M	Q,K	I,E,V	0.011	0.741	A68, A69, B18, B27, B37, C03, C06

# sites without HLA associated polymorphisms.

*: sites detected with diversifying selection model (D); *WT,* wild type states are amino acids for which there is a significant difference between null and alternate models in a likelihood ratio test (boldface indicates state with largest log likelihood); E, escape; R, reversion; *t_c_*, proportion of time spent in class c (Figure 1) for wild type state with the largest likelihood.

## Discussion

HIV-1 evolves rapidly and under strong selective pressure. Although purifying selection acting on coding regions is important for preserving protein functions [55], positive selection has been shown [9], [36]–[40] to play an important role in shaping HIV-1 genetic diversity. In particular, sites involved in escape from host immune responses, either due to CTLs or neutralizing antibodies, have frequently been reported to be under strong selection pressure [1],[6],[10],[21],[56]. Because the host immune response is highly polymorphic, viral sequences sampled from multiple hosts reflect an ongoing history of adaptation to successive host immune responses and to successive responses mounted by the adaptive immune system within individual hosts. Some mutations that facilitate escape from immune responses occur at sites that are functionally constrained [2]. These typically incur a fitness cost to the virus, and thus come under selection to revert to the wild type amino acid upon transmission to a host without the immune response [32]–[35],[57]. Escape mutations that occur at sites that are functionally unconstrained, or for which compensatory mutations [22] fully offset the fitness cost, will not experience selection for reversion. In this case the escape state may persist over time, and become fixed [58], in the absence of further immune responses targeting the same site.

In general, escape mutations in a viral epitope could be classified according to whether they prevent recognition of the epitope by a specific clonal population of immune cells or whether they interfere with the processing or presentation of the epitope. The latter can cause permanent escape from immune responses targeted against a specific epitope within an individual, while the former may be targeted again by a future immune response in the same individual. Successive escape mutations, particularly when they occur at no great cost to the virus in terms of fitness are likely to result in a pattern of diversifying selection, where all of the possible non-synonymous substitutions at a site are affected by positive selection. By contrast, mutations that prevent epitope presentation and which revert in the absence of the immune response would be likely to fit a pattern of amino acid toggling. Much of the adaptive evolution in viral coding sequences is likely to be a consequence of the adaptive immune response, but *a priori* there is no way to know whether this primarily involves immune escape and reversion at functionally constrained sites or diversifying selection at less constrained sites.

We introduce a model of toggling selection that seeks to model the process of immune escape and reversion at constrained sites. Our model differs from standard codon models in the manner in which equilibrium codon frequencies are included. Typically, codon frequencies are estimated from the alignment and the relative rates of substitution between codons are a product of these alignment-wide codon frequencies and exchangeability parameters that depend on the nature of the mutation. Advances in phylogenetic modeling have allowed frequency parameters to vary between sites [59]. Our model of immune escape and reversion similarly allows sites to have independent codon frequency parameters; however, we do this through the introduction of just two frequency parameters (rather than the 19 or 60 free parameters required per site if the frequency of each amino acid or codon was free to vary independently at each site).

Using simulation we find, unsurprisingly, that this model is more efficient at detecting toggling than a model of diversifying selection, while the opposite is the case for sites evolving under diversifying selection. Interestingly, when we apply both models to coding sequence data from HIV-1, the toggling model detects a larger number of positively selected sites. Furthermore, positively selected sites detected using either or both models more often provide a better fit, using AIC [54], to the toggling selection model than to the diversifying selection model (Table 3). Taken together, these observations suggest that a large proportion of positive selection in HIV-1 consists of escape and reversion at functionally constrained sites, and that the toggling model is a better description of adaptive evolution in HIV-1 than standard models of diversifying selection. Because diversifying selection and toggling selection are consistent with distinct biological scenarios, models that attempt to fit the specific characteristics of each scenario are of value. This is of particular relevance since the toggling selection model displays significantly improved power to detect escape and reversion, a process which is important because it characterizes immune responses that are effective in controlling viral replication. The significance of sites that display rapid immune escape and reversion is evident from the fact that these sites are often the targets of HLA alleles that confer improved disease prognosis [13],[18],[35],[60].

**Figure 5:**
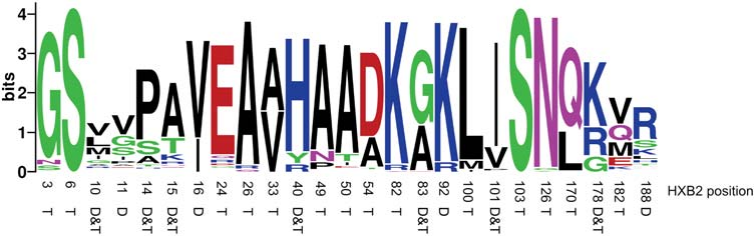
Amino acid diversity at positively selected sites. Amino acid sequence logos [73] of positively selected sites, diversifying selection (D), toggling (T), or both (D&T), indexed by HXB2 position in *nef* are shown.

Some overlap occurs between sites detected with a diversifying selection and toggling selection models (Figure 5, Figure S3, Figure S4); however, in all genes, approximately as many or more sites are detected using the toggling model than the diversifying selection model (Figure S3). The fact that many sites detected using the toggling selection model are not detected using the diversifying selection model (and vice versa) suggests that the models are not redundant and are sensitive to different trends in the data. Both *nef* and *env* have significantly more sites detected with a toggling model than with a diversifying selection model. Amino acid diversity at positively selected sites (detected with either the diversifying or toggling selection models) is generally lower (for example *nef*, Figure 5) than one would expect for diversifying selection. The toggling model detects positive selection at a greater proportion of these low amino acid diversity sites, although some sites with low diversity are detected with the diversifying selection model and not the toggling model, for example *nef* site 16. At this site toggling selection is not detected when averaging over all potential wild-type amino acids (Equation 3), but there is significant evidence for selection with one wild type amino acid (valine, *P*<0.001), exemplifying the loss in power associated with summing over the uncertainty of the wild-type amino acid.

Interestingly, some sites at which no non-synonymous substitutions are observed (6, 103; Figure 3) show significant evidence of toggling. Serine is encoded by islands of codons (TCN and AGY). At both site 6 and 103 serine is encoded by TCN and AGY codons, implying the occurrence of non-synonymous substitutions from serine to another amino acid and back to serine, despite the fact that no other amino acids are observed at this site. This is consistent with immune escape and reversion, in which escape occurs from serine to another amino acid followed by reversion to an alternative encoding of serine, at a remove of two substitutions from the original codon. However, it is also possible that mutations between these sets of serine codons could occur as a single event through doublet mutations [61], which are not considered in our model and are a potential source of false positive results with most models of positive selection.

The toggling selection that we observed in *nef* is consistent with previous studies demonstrating a high density of HLA-associated polymorphisms [4],[25], and a high level of immunogenicity and density of epitopes [62] in this gene. Several of the sites identified as toggling (Table 4) map to within HLA epitopes for which there was a significant association between the presence of a viral polymorphism and presence or absence of an HLA allele, consistent with immune escape and reversion [4]. For example, site 83 has significant association with several HLA alleles (Table 4; [4]). We find evidence for toggling with either of the two previously identified reversion mutations as wild type, and overall a general pattern of escape and reversion across the phylogenetic tree (Figure S5). Furthermore, the inferred times spent in the wild type and single step escape states are consistent with toggling, and indicate either the strength of selection or frequency of the host immune response. Several sites spend a high proportion of time in the wild type state (Table 4), consistent with a low frequency of the immune response in the population, or strong selection to revert upon transmission to a new host. Sites at which time is equally distributed between the wild type and escape states (Table 4) suggest either an intermediate frequency of the immune response, or reduced selection pressure to revert. Finally, sites with little time in either the wild type or escaped states are likely to indicate either misidentification of the wild type state or that the evolution at the site does not fit well with a model of escape and reversion from a single immune response with a fixed wild type, or most fit state, in the absence of the immune response. In Table 4, sites such as 83, where the same amino acid occurs as an escape and a reversion, can be explained as resulting from multiple overlapping epitopes, such that an amino acid can be a wild type or an escape state, depending on the HLA genotype of the host [4]. This is particularly common in *nef* (Figure S4, [4]). Although our model, and the simulations we conducted, assumes a single wild type amino acid at each site, we still detect selective pressure at sites that have multiple potential wild type states (Table 4).

In *env*, which is targeted by both cellular and humoral immune responses [10] we detect significant evidence for toggling with multiple potential wild type states (*P* = 0.02), at an N-linked glycosylation site (N392A), but no evidence for diversifying selection at this site (*P* = 0.92). N-linked glycosylation sites are associated with binding of carbohydrates that may either be recognized by specific antibodies [63] or assist in the evasion of host antibodies through the formation of a glycan shield [6]. In particular, asparagine (N392A) facilitates the binding of the monoclonal antibody, 2G12, to gp120 [63],[64], which suggests asparagine (N) is a susceptible state, but only in the presence of 2G12. This site provides a good example of conflicting selective pressure, since asparagine is susceptible in the presence of 2G12, but may represent an escape state in its absence, by contributing to evasion of antibody responses through the formation of a glycan shield [6].

We find a smaller proportion of positively selected sites favoring the toggling selection model for *gag* than for other coding regions. This is somewhat surprising since broad *gag* CTL responses control viremia [16],[65], several known protective HLA alleles target *gag* [13], and fitness costs of many of the escape mutations in *gag* are substantial [15], [33]–[35]. A recent study identified escape and reversion mutations through the mapping of polymorphisms (observed longitudinally within acutely-infected individuals) to epitopes in a pre-defined list of HLA-associated polymorphisms [25]. Results indicate an early CTL response biased towards protective HLA alleles (B*13, B*51, B*57, B*5801), and for which mutations in *gag* were reverting most rapidly [25]. Similarly, the detection of escape and reversion mutations through HLA-associated polymorphisms [26] also found strong evidence for reverting mutations in *gag*.

To understand the lack of support for toggling selection in *gag* we investigated a well-characterized *gag* epitope (TW10) which is targeted by a protective HLA allele (B*57) [17],[34]. We detected only diversifying selection at the site of the common TW10 escape mutation (T242N). To determine why this well-characterized site of escape and reversion is detected by a diversifying selection model and not by the toggling selection model we mapped the occurrence of wild type, neighboring and multi-step codon states for T242N to the phylogeny estimated from *gag* sequences (Figure 4), taking threonine, which is known to be the susceptible amino acid [17],[34] as the wild type state. Escape mutations are evident at terminal branches; however there is no example on the tree of a case in which the wild type amino acid appears within a clade of escape amino acids (which would point to reversion of an escape mutant to wild type). The likely reason for this is that escape and reversion happen sufficiently rapidly that the amino acids observed in neighboring sequences on the tree are uncorrelated. In such a case, we are unlikely to infer reversion to wild type, particularly because a given HLA allele occurs in only a small minority of individuals and most clades of sequences will be dominated by sequences from individuals without the HLA allele. Consequently multiple independent escapes will be a more parsimonious explanation of the data than escape followed by reversion.

This is consistent with the much lower power to detect high rates of toggling simulated along typical HIV-1 trees with long terminal branches compared to the power to detect the same rate of toggling on trees with exponentially distributed branch lengths (Table 2, Figure S1). Thus failure to detect toggling selection at site 242 of *gag* is likely the result of both rapid reversion at this site [25], and long terminal branches. Sites that exhibit such rapid escape and reversion are likely to be relatively easily detected through association of HLA alleles with viral sequence polymorphisms [4],[24]. However, we note that we compare previously detected HLA associations using a substantially larger dataset [4], to sites detected as toggling in this analysis, in which only 100 sequences were used. Association methods will perform well when the HLA allele tested is common, but will lose power when multiple conflicting rare HLA alleles exert conflicting selective pressures. The use of a phylogenetic model to detect positive selection associated with host-immune response allows for the identification of sites at which multiple contrasting selective pressures are exerted by immune responses of low to intermediate frequencies.

We found evidence of a large number of coding sites in HIV-1 where the amino acid diversity is limited, yet there is strong positive selection pressure. Intuitively, it is easy to see why a model that takes account of this should have more power to detect selection than models of diversifying selection. With a diversifying selection model a mutation away from the wild type amino acid followed by a reversion to wild type is treated the same as any other pair of non-synonymous substitutions. Whenever this pattern is observed, the toggling model accumulates much more evidence for positive selection pressure, because in the absence of selection the second mutation should be far more likely to result in a different amino acid than a reversion to the original (random point mutations in a codon can result in any one of approximately eight different amino acids, depending on the specific codon). Using the synonymous substitution rate as a proxy for neutrality, we have set up a model that can detect when this toggling occurs at a greater rate than we would expect for a site that is neutral or evolving under purifying selection. We expect this model to be applicable to other host-pathogen systems in which escape from immune responses can occur at a cost to the pathogen. There is some evidence that this is likely to apply in the context of influenza A, for example, which is targeted by both cellular [66] and humoral [67],[68] host immune responses, and appears to toggle between alternate states at some sites [67]. Furthermore, mutations that confer drug resistance to pathogens may do so at a cost to the pathogen [69],[70]. Samples derived from drug treated and untreated individuals are likely to exhibit a pattern of toggling if the drug resistant pathogen reverts to a stable most fit state, in the absence of the drug. In the case of drug resistance mutations, models that take account of the treatment status of the sequence are likely to provide more power to detect the evolution of resistance mutations [71].

Sites that experience strong selection but limited diversity point to the limits of viral evolution. Despite coming under pressure to change at these sites, the virus continuously returns to a single or small number of fit states, which at many such sites appear to remain relatively stable over the course of viral evolution. Distinguishing sites at which there is strong positive selection to revert to the wild type is relevant for vaccine design. Vaccines targeting these sites may allow for better control of viremia by reducing replicative fitness of the viral population resulting in slower disease progression [5],[72].

## Supporting Information

Figure S1 Amino acid toggling and tree shape. Toggling was simulated either (A) along a random tree in which branch lengths were drawn from an exponential distribution (mean = 0.05), or (B) along an HIV-1 tree estimated from published nef sequence data [4].
(0.02 MB PDF)

Figure S2 Effect of tree shape on power to detect positive selection. Simulated data was used to construct ROC plots of the effects of tree shape on performance of both models. (A) Diversifying selection. (B) Positive selection (diversifying selection and toggling). (C) Amino acid toggling only.
(0.24 MB PDF)

Figure S3 Number of sites under positive selection in real HIV-1 data. Number of positively selected sites detected using a standard diversifying selection model (D) compared to a toggling model (T) for each of four HIV-1 genes; (A) pol, (B) nef, (C) gag, (D) env. Counts indicate numbers of positively selected sites identified with each method, the number of shared sites, and the number of selectively neutral sites.
(0.26 MB PDF)

Figure S4 HLA epitope maps of sites under positive selection. Positively selected sites identified using a standard diversifying selection model (D) or the toggling model (T) in (A) nef, (B) env, (C) gag, (D) pol. Sites unique to each model are shown as open circles, whereas shared sites are indicated with triangles. Optimal CTL epitopes (http://www.hiv.lanl.gov/content/index) are shown as solid black lines. Positively selected sites mapping within epitopes are shown in red. Overlapping peptides for which there is a significant association between the recognition of a peptide and expression of an HLA class I allele in the gag study [53] are shown as red lines. Recombination breakpoints (bp), identified using GARD [51], demarcate gene regions for which independent phylogenetic trees were estimated.
(0.05 MB PDF)

Figure S5 Mapping of wild type and escape mutations to phylogeny. Mapping of codon states to terminal branches for nef site 83. Branches are colored according to codon category, c (Figure 1), Taxon labels are accession_codon. Tree is rooted with subtype B HIV-1 sequence.
(0.24 MB PDF)

Table S1 Comparison of the area under the ROC curves shown in Figure 3.
(0.03 MB DOC)
