# Supplementary figures and images for: Frequent Toggling between Alternative Amino Acids Is Driven by Selection in HIV-1
Source: PLoS Pathog. 2008 Dec 19;4(12):e1000242. doi: 10.1371/journal.ppat.1000242 (PMC2592544; doi:10.1371/journal.ppat.1000242)

A

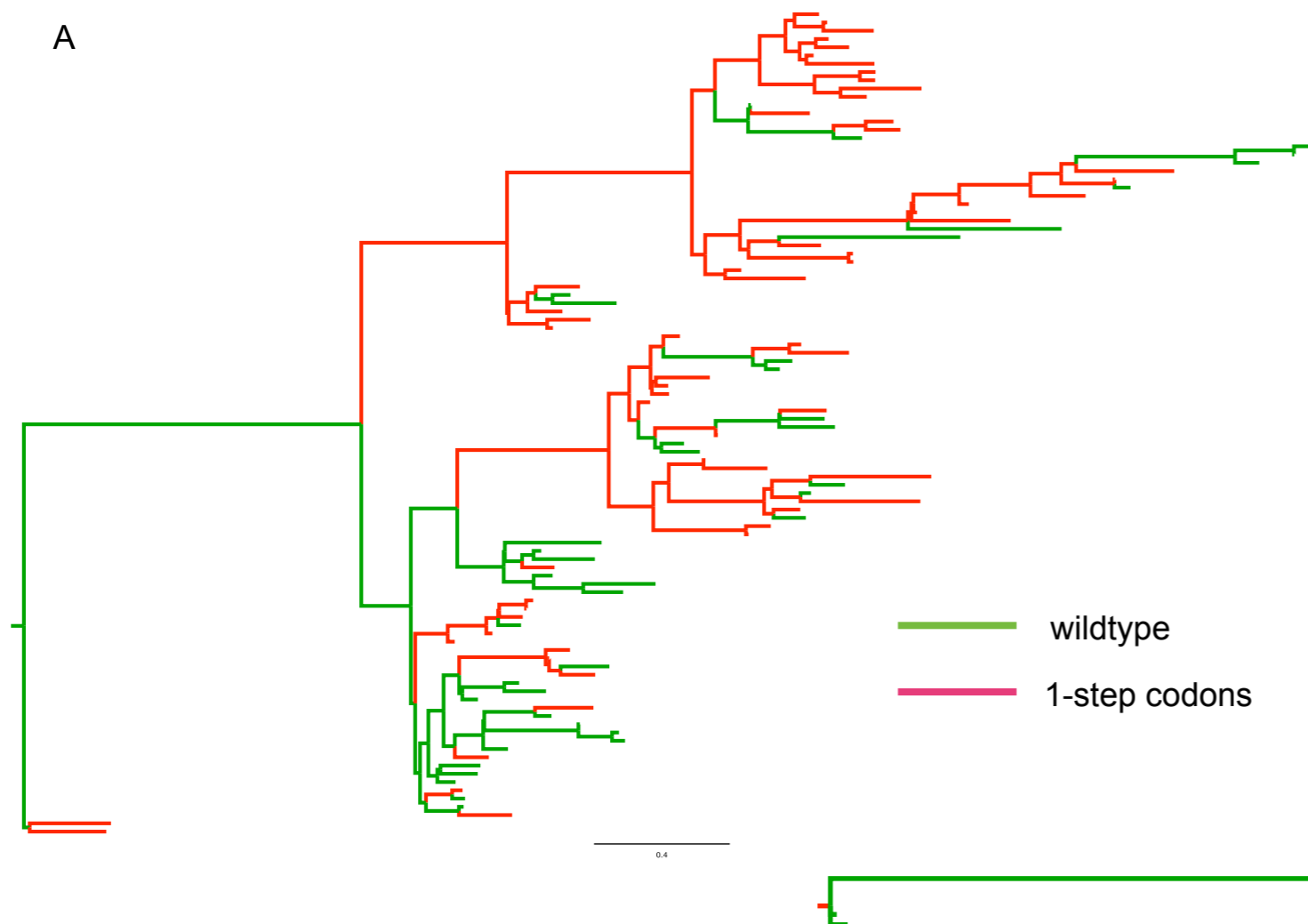

B

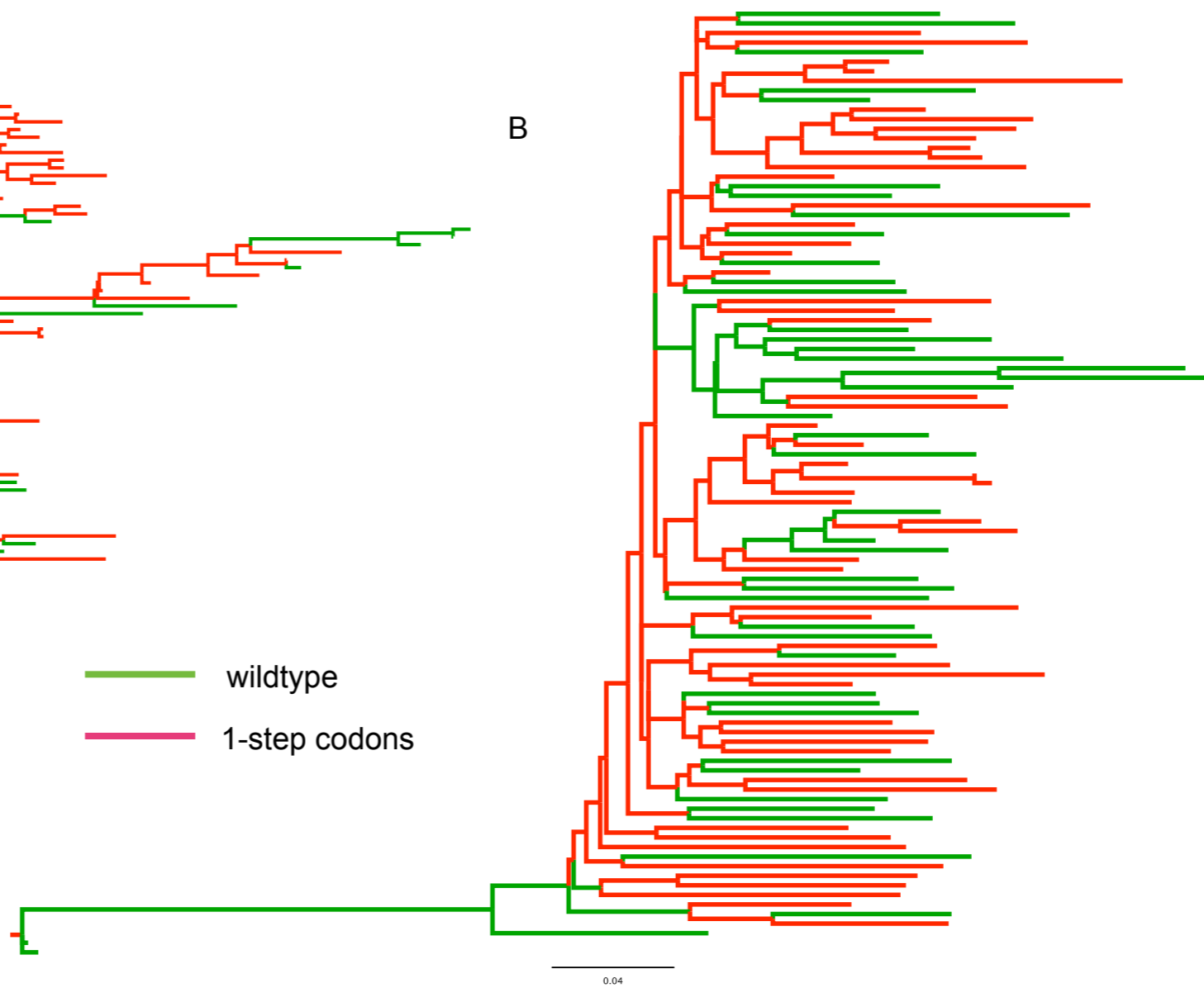

Supplement: Figure S1 — Amino acid toggling and tree shape. Toggling was simulated either (A) along a random tree in which branch lengths were drawn from an exponential distribution (mean = 0.05), or (B) along an HIV-1 tree estimated from published nef sequence data [4]. (0.02 MB PDF) [file ppat.1000242.s001.pdf]

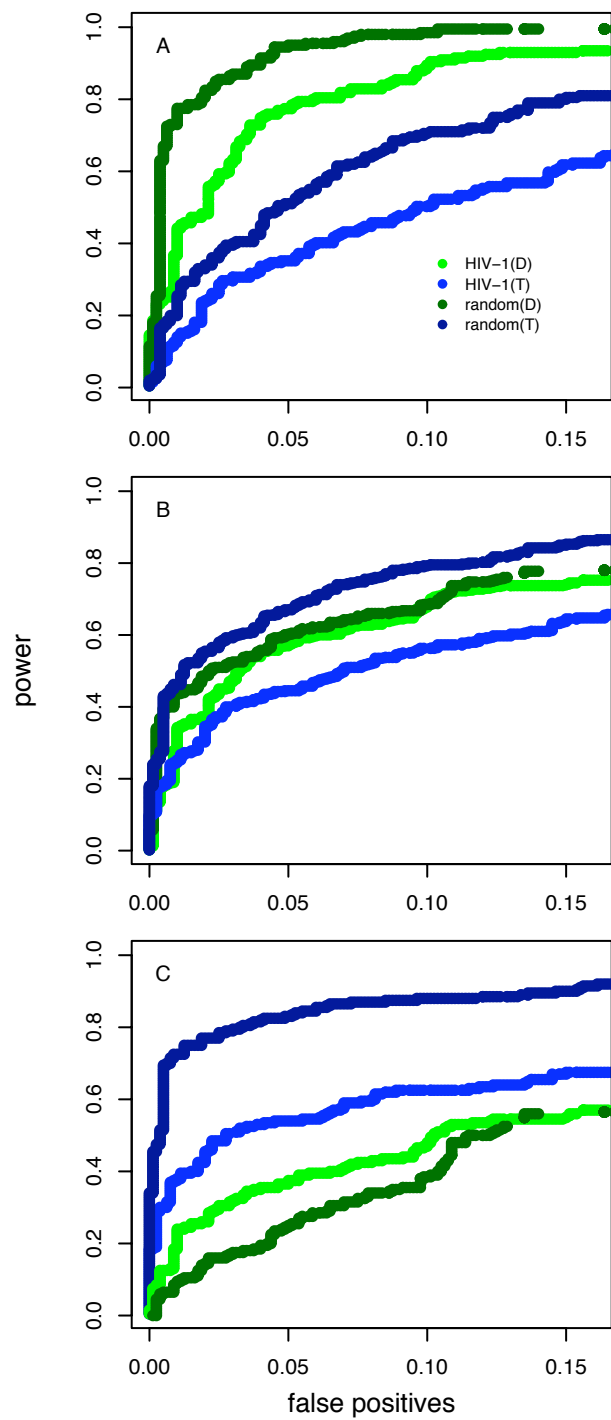

Supplement: Figure S2 — Effect of tree shape on power to detect positive selection. Simulated data was used to construct ROC plots of the effects of tree shape on performance of both models. (A) Diversifying selection. (B) Positive selection (diversifying selection and toggling). (C) Amino acid toggling only. (0.24 MB PDF) [file ppat.1000242.s002.pdf]

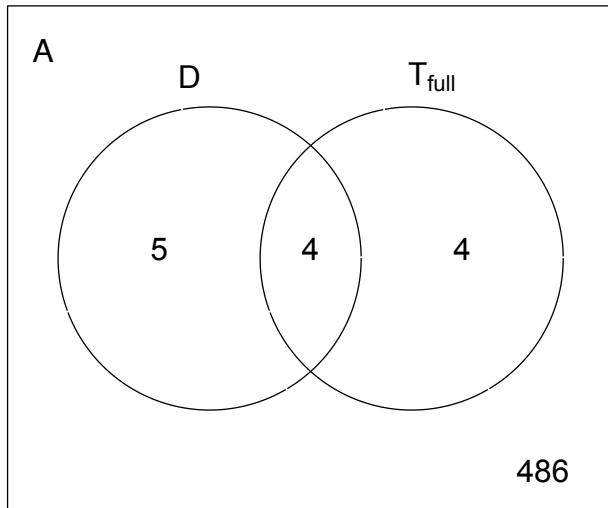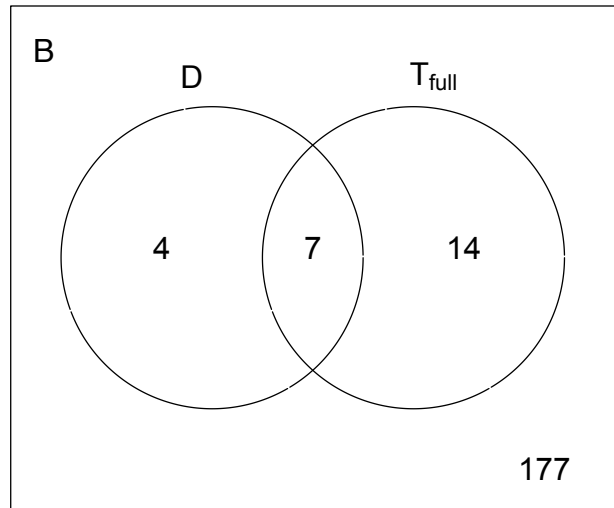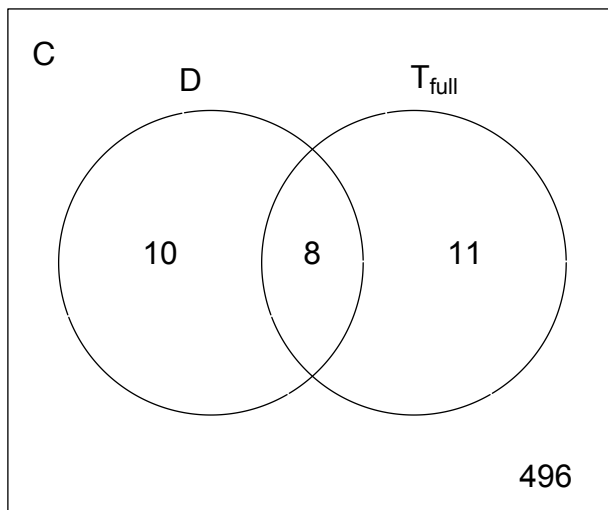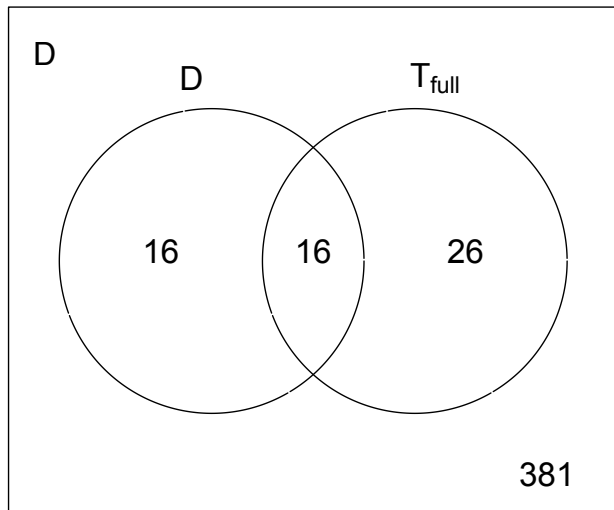

Supplement: Figure S3 — Number of sites under positive selection in real HIV-1 data. Number of positively selected sites detected using a standard diversifying selection model (D) compared to a toggling model (T) for each of four HIV-1 genes; (A) pol, (B) nef, (C) gag, (D) env. Counts indicate numbers of positively selected sites identified with each method, the number of shared sites, and the number of selectively neutral sites. (0.26 MB PDF) [file ppat.1000242.s003.pdf]

A

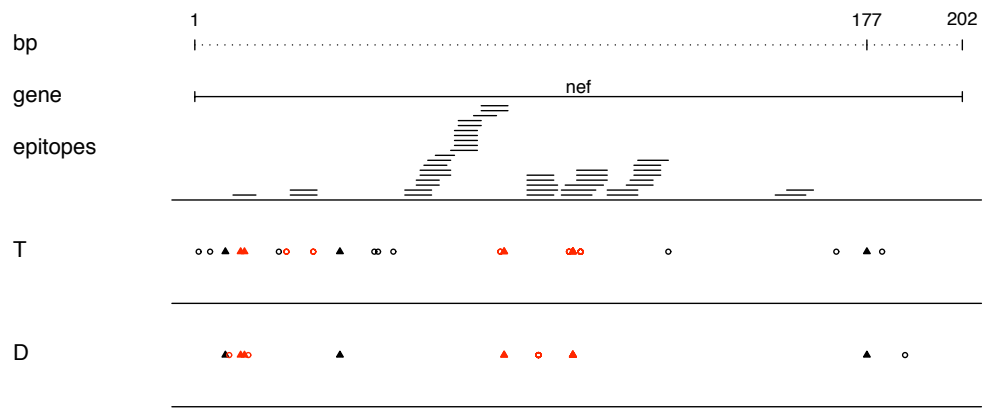

B

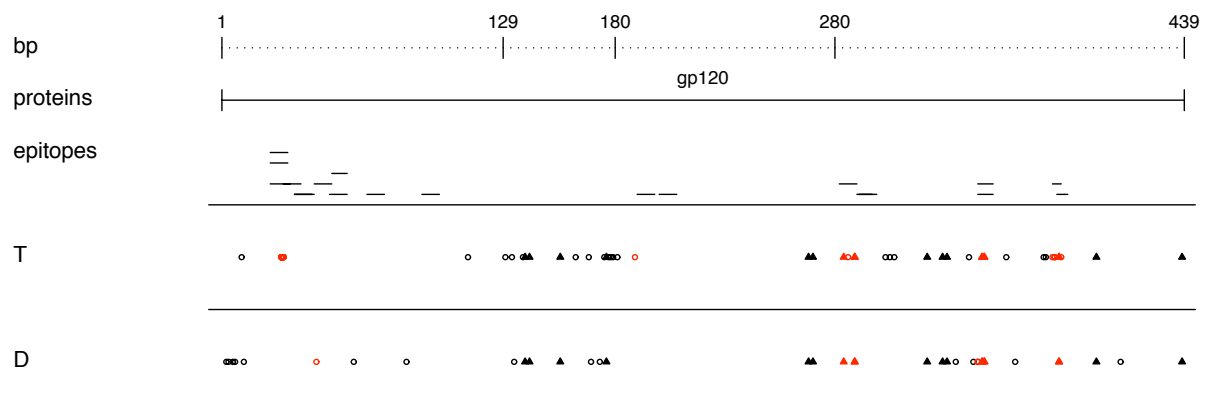

C

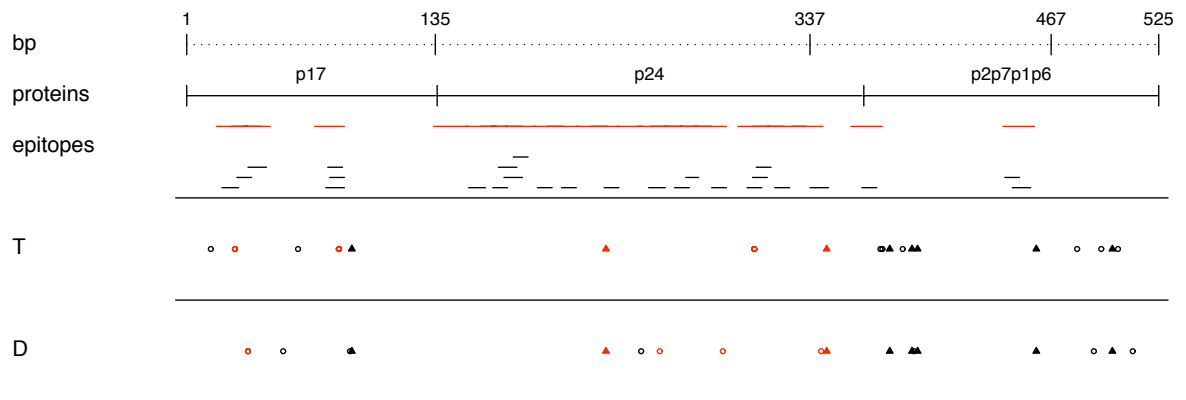

D

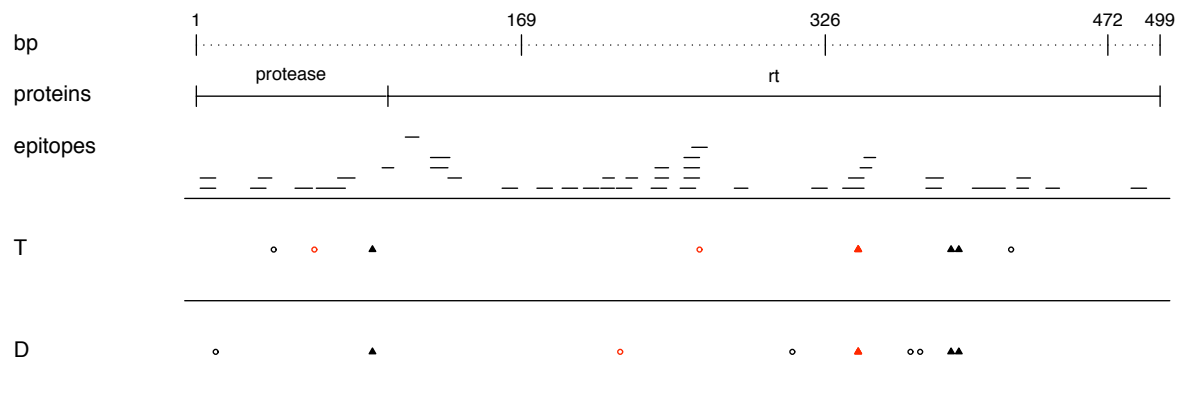

Supplement: Figure S4 — HLA epitope maps of sites under positive selection. Positively selected sites identified using a standard diversifying selection model (D) or the toggling model (T) in (A) nef, (B) env, (C) gag, (D) pol. Sites unique to each model are shown as open circles, whereas shared sites are indicated with triangles. Optimal CTL epitopes (http://www.hiv.lanl.gov/content/index) are shown as solid black lines. Positively selected sites mapping within epitopes are shown in red. Overlapping peptides for which there is a significant association between the recognition of a peptide and expression of an HLA class I allele in the gag study [53] are shown as red lines. Recombination breakpoints (bp), identified using GARD [51], demarcate gene regions for which independent phylogenetic trees were estimated. (0.05 MB PDF) [file ppat.1000242.s004.pdf]

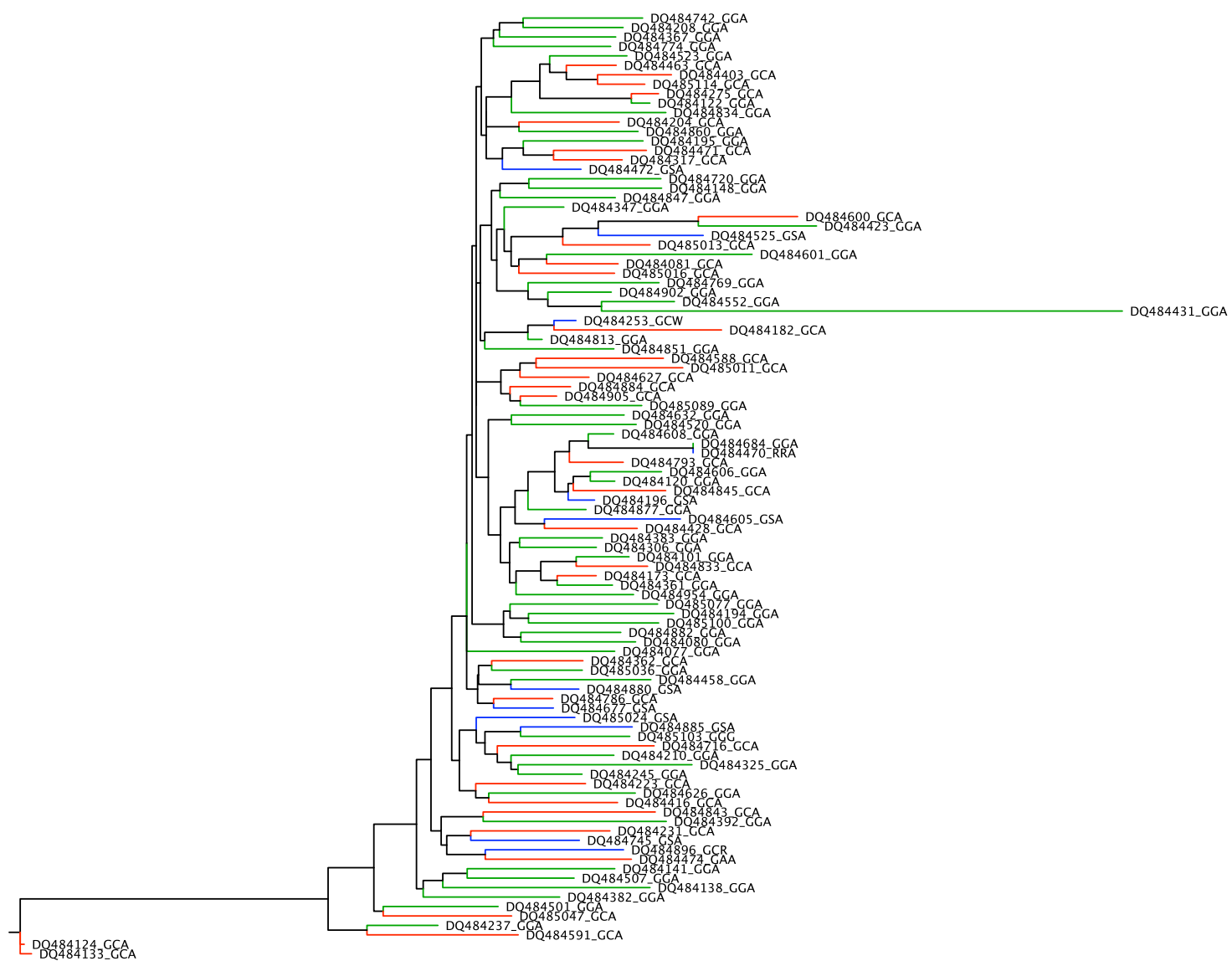

0.06

Supplement: Figure S5 — Mapping of wild type and escape mutations to phylogeny. Mapping of codon states to terminal branches for nef site 83. Branches are colored according to codon category, c (Figure 1), Taxon labels are accession_codon. Tree is rooted with subtype B HIV-1 sequence. (0.24 MB PDF) [file ppat.1000242.s005.pdf]
