# Supplementary material for: Frequent Toggling between Alternative Amino Acids Is Driven by Selection in HIV-1
Source: PLoS Pathog. 2008 Dec 19;4(12):e1000242. doi: 10.1371/journal.ppat.1000242 (PMC2592544; doi:10.1371/journal.ppat.1000242)
Supplement: Table S1 — Comparison of the area under the ROC curves shown in Figure 3. (0.03 MB DOC) [file ppat.1000242.s006.doc]

**Table S1**. Comparison of the area under the ROC curves shown in Figure 3

|  | **Diversifying selection** | | | **Positive selection** | | | **Toggling** | | |
| --- | --- | --- | --- | --- | --- | --- | --- | --- | --- |
| ***ρ*** | ***AUC_D_*** | ***AUC_T_*** | ***P*** | ***AUC_D_*** | ***AUC_T_*** | ***P*** | ***AUC_D_*** | ***AUC_T_*** | ***P*** |
|  |  |  |  |  |  |  |  |  |  |
| 2 | **0.738** | 0.687 | 0.024 | 0.655 | 0.669 | 0.484 | 0.573 | **0.653** | 0.006 |
| **3** | **0.903** | 0.790 | < 0.001 | 0.751 | 0.773 | 0.228 | 0.600 | **0.759** | < 0.001 |
| **4** | **0.931** | 0.799 | < 0.001 | 0.789 | 0.803 | 0.438 | 0.649 | **0.810** | < 0.001 |
| **5** | **0.982** | 0.829 | < 0.001 | 0.850 | 0.818 | 0.057 | 0.720 | **0.810** | < 0.001 |
|  |  |  |  |  |  |  |  |  |  |
| *AUC_D_* = Area under diversifying selection model ROC curve, *AUC_T_* = area under toggling model ROC curve, *ρ* = non-synonymous to synonymous rate ratio associated with wildtype amino acid, *P* = *p*-value of test. Boldface type indicates models for which AUC’s which are significantly greater. | | | | | | | | | |
